# A newly discovered glycosyltransferase gene *UGT88A1* affects growth and polysaccharide synthesis of *Grifola frondosa*

**DOI:** 10.1007/s00253-024-13062-0

**Published:** 2024-02-29

**Authors:** Jian Li, Bao-xin Wang, Jie Zhang, Na Han, Shu-ting Liu, Wen-ji Geng, Shi-ru Jia, Yan-ru Li, Quan Gan, Pei-pei Han

**Affiliations:** State Key Laboratory of Food Nutrition and Safety, Key Laboratory of Industrial Fermentation Microbiology, Ministry of Education, College of Biotechnology, Tianjin University of Science and Technology, Tianjin, 300457 People’s Republic of China

**Keywords:** *UGT88A1*, *Grifola frondosa* polysaccharides, Polysaccharide synthesis, Cell wall integrity, Antioxidant activity

## Abstract

**Abstract:**

*Grifola* *frodosa* polysaccharides, especially β-D-glucans, possess significant anti-tumor, antioxidant and immunostimulatory activities. However, the synthesis mechanism remains to be elucidated. A newly discovered glycosyltransferase UGT88A1 was found to extend glucan chains in vitro. However, the role of UGT88A1 in the growth and polysaccharide synthesis of *G. frondosa* in vivo remains unclear. In this study, the overexpression of *UGT88A1* improved mycelial growth, increased polysaccharide production, and decreased cell wall pressure sensitivity. Biomass and polysaccharide production decreased in the silenced strain, and the pressure sensitivity of the cell wall increased. Overexpression and silencing of *UGT88A1* both affected the monosaccharide composition and surface morphology of *G. frondosa* polysaccharides and influenced the antioxidant activity of polysaccharides from different strains. The messenger RNA expression of glucan synthase (*GLS*), UTP-glucose-1-phosphate uridylyltransferase (*UGP*), and UDP-xylose-4-epimerase (*UXE*) related to polysaccharide synthesis, and genes related to cell wall integrity increased in the overexpression strain. Overall, our study indicates that *UGT88A1* plays an important role in the growth, stress, and polysaccharide synthesis of *G. frondosa*, providing a reference for exploring the pathway of polysaccharide synthesis and metabolic regulation.

**Key points:**

*•UGT88A1 plays an important role in the growth, stress response, and polysaccharide synthesis in G. frondosa.*

*•UGT88A1 affected the monosaccharide composition, surface morphology and antioxidant activity of G. frondosa polysaccharides.*

*•UGT88A1 regulated the mRNA expression of genes related to polysaccharide synthesis and cell wall integrity.*

**Supplementary Information:**

The online version contains supplementary material available at 10.1007/s00253-024-13062-0.

## Introduction

*Grifola frondosa* is an edible medicinal fungus. Numerous studies have shown that *G. frondosa* polysaccharides possess various biological activities and broad application prospects in anti-tumor, anti-oxidation, anti-radiation, and improving immunity (Mao et al. 2014; Masuda et al. 2009). *Grifola frondosa* glucans mainly consist of β-(1 → 3, 1 → 6)-D-glucan and can be divided into intracellular polysaccharides (IPSs) and extracellular polysaccharides (EPSs), which have differences in structure, monosaccharide composition, and activity (He et al. 2017). *Grifola frondosa* glucans can be further purified using a cellulose anion-exchange column to obtain neutral and acidic polysaccharides (Wang et al. 2021). Studies have confirmed a correlation between monosaccharide composition and polysaccharide activity. For example, polysaccharides from *Tremella mesterica* are rich in D-mannose (D-Man) and enhance the immune system by stimulating macrophage receptors (Chabot et al. 2001); polysaccharides rich in L-fucose (L-Fuc) show better anti-cancer and anti-inflammatory activities (Cescutti et al. 2005), and L-arabinose (L-Ara) plays an important role in scavenging free radicals by polysaccharides (Lo et al. 2007). Polysaccharide synthesis and monosaccharide composition in *G. frondosa* are involved in the synergistic regulation of numerous genes. Therefore, the identification of genes related to polysaccharide synthesis is a key research objective to achieve better and more effective applications of polysaccharides in this field.

Yang et al. (2023) summarized the recent of UDP- glycosyltransferases, including their structures, functions, and catalytic mechanism, especially also in edible fungi. Different types of glycosyltransferases catalyze the transfer of sugars to a variety of receptor molecules such as uridine diphosphate (UDP)-glucose (Glc), UDP-galactose (Gal), UDP-xylose (Xyl), and other non-sugar molecules, and these glycosyltransferases belong to a large family involved in sugar metabolism (Ross et al. 2001). Researchers have established a preliminary approach to fungal polysaccharide synthesis pathways based on the identification of intermediates and key enzymes by researchers (Ji et al. 2015; Zan et al. 2020a). From the established pathways, polysaccharide synthesis requires the assembly of multiple monosaccharide precursors via glucan synthase (Cui et al. 2019), whereas monosaccharide precursors require catalysis by multiple enzymes, including phosphoglucose translocase to catalyze Glc-6-P to Glc-1-P (Zhu et al. 2016), followed by UDP-glucose pyrophosphorylase catalyzing the production of UDP-Glc (Li et al. 2015), which is the direct precursor of polysaccharide synthesis. Synthetic pathways of other monosaccharide precursors such as deoxythymidine diphosphate (dTDP)-rhamnose (Rha), UDP-Gal, and guanosine-5′-diphosphate (GDP)-Man have also been reported (Peng et al. 2016; Zhou et al. 2014).

UDP-glycosyltransferase 88A1 (UGT88A1) from the glycosyltransferases family (GTs; EC 2.4.x.y) is a sugar-dependent glycosyltransferase, that was first expressed and characterized in *Arabidopsis thaliana* (Weng et al. 2019). UGT88A1 is believed to be involved in the enzymatic synthesis of quercetin-4′-*O*-glucoside in *A. thaliana*. However, a recent study showed that the glycosyltransferase UGT88A1 from *G. frondosa* showed superior activity in extending oligosaccharide chains in vitro, with relatively high polymerization of oligosaccharide chains as the acceptor when using UDP-Glc as a donor (Yyl et al. 2022). Similarly, the enzyme UGT1 of *Aureobasidium melanogenum* P16 which donors UDP-Glc can catalyze the transfer of glucosyl from UDP-glucose to oligosaccharides, polysaccharides, lipids, proteins, and other macromolecules in the yeast cells. After completely removing the *UGT1* gene, the mutant produced 27.7 ± 3.1 g/L of pullulan polysaccharides, which was less than the wild-type strain producing 63.38 ± 2.0 g/L of pullulan polysaccharides (Chen et al. 2017). However, the effect of UGT88A1 on polysaccharide synthesis in *G. frondosa* in vivo remains unclear. In our previous study, farnesol was added to *G. frondosa* medium which increased EPS production during liquid fermentation and affected its physicochemical properties (Wang et al. 2021). Farnesol is reported as a quorum sensing molecule in fungi, and it can affect mycelium formation, biofilm formation, oxidative stress, and regulate drug efflux (Jabra-Rizk et al. 2006). From the transcriptome data of this model, it was found that *UGT88A1* was significantly up-regulated in high-yield strains, which may contribute to the synthesis and physicochemical properties of polysaccharides. Overexpression is a commonly used method for identifying gene function and has been successfully applied in *G. frondosa* (Zan et al. 2020b). RNA interference (RNAi) is a convenient method for reverse genetics. Exogenous or endogenous double-stranded RNA (dsRNA) is cleaved into 21–23 nt small interfering RNA (siRNA) molecules by RNase-III like enzymes (DICER). The siRNA is then incorporated into the RNA-induced silencing complex as a guiding molecule to target the homologous messenger RNA (mRNA) to reduce gene expression. In fungi, this mechanism was first discovered in *Neurospora crassa* (Romano and Macino 1992). This method was first developed and used for *G. frondosa* by Sato et al. (2014), and has since been widely applied to elucidate gene functions in organism (Jiang et al. 2022; Zan et al. 2020a, b). In the present study, the effects of this gene on growth, mycelial elongation, polysaccharide synthesis, monosaccharide composition, and polysaccharide antioxidant activity of *G. frondosa* were investigated via overexpression and silencing.

## Materials and methods

### Strain and culture conditions

*Grifola frondosa* CGMCC 5.404 was obtained from the Chinese General Microbial Strains Conservation Center (CGMCC, Beijing, China). The strain was stored at 4 °C on potato dextrose agar (PDA) medium consisting of 200 g/L of potato, 20 g/L of glucose, 5 g/L of tryptone, 2 g/L of KH_2_PO_4_, 1.0 g/L of MgSO_4_·7H_2_O, 0.02 g/L of vitamin B_1_ (VB_1_), and 20 g/L of agar, slanted, and subsequently subcultured every 4–6 weeks at 28 °C. The regeneration medium (MYG) used for *G. frondosa* protoplast regeneration and screening of transformants contained 200 g/L of potato, 20 g/L of glucose, 5 g/L of tryptone, 0.6 M of mannitol, 2 g/L of KH_2_PO_4_, 1 g/L of MgSO_4_·7H_2_O, and 0.02 g/L of vitamin B_1_ (top layer with 10 g/L of agar, bottom layer with 20 g/L of agar and 100 μg/mL hygromycin B). The mycelium was macerated and cultured in a 250-mL grooved Erlenmeyer flask containing 100 mL of the medium (40 g/L of glucose, 5 g/L of tryptone, 2 g/L of KH_2_PO_4_, 1 g/L of MgSO_4_·7H_2_O, 0.02 g/L of VB_1_) at 160 rpm in a shake incubator at 28 °C for 5 days. Inoculum (10%) was inoculated in a 250-mL grooved Erlenmeyer flask containing 100 mL of fermentation medium (22 g/L of glucose, 3 g/L of tryptone, 1.2 g/L of KH_2_PO_4_, 0.8 g/L of MgSO_4_·7H_2_O, 0.12 g/L of VB_1_) and cultured at 160 rpm at 28 °C for 7 days. *Escherichia coli* DH5α (Takara, Osaka, Japan) was used for vector construction and sub-cloning experiments.

### Cloning and sequencing of the *UGT88A1* coding region

The putative *G. frondosa* gene (*GFUGT*) was retrieved with the access number OBZ67170.1 based on the transcriptome data and the whole genome sequence (accession NO.: ASM168373v1) of strain 9006–11 published by the National Center for Biotechnology Information (NCBI). The total RNA of *G. frondosa* was extracted from fresh mycelia using a Fungal RNA extraction kit (Omega, Mogadore, OH, USA). The total RNA was transcribed to a cDNA library using the PrimeScript™ RT Kit with DNAse (Takara, Osaka, Japan). The coding sequence of the *GFUGT* fragment was amplified from a cDNA library using the primers GFUGT-F/R(shown in Supplemental Table S1). The gel product was purified and recovered with a Tiangen DP219 kit (Tiangen, Beijing, China), sequenced by GENEWIZ Co., Ltd. (Suzhou, China) to proof the nucleotide sequence again, and aligned with the blast-n program (https://blast.ncbi.nlm.nih.gov/Blast.cgi).

### Construction of plasmids and transformation of *G. frondosa*

The dual promoter RNAi plasmid pAN7-iUGT was constructed by referring to Mu et al. (2012) and Sato et al. (2014). Briefly, the conserved functional sequence of the glyceraldehyde-3-phosphate dehydrogenase gene promoter (*gpd*, 956 bp) and the functional region of glyceraldehyde-3-phosphate dehydrogenase promoter (*gpd*A, 977 bp) from *Aspergillus nidulans* were amplified from the *G. frondosa* genome and the pAN7-1 plasmid (Punt et al. 1987), respectively. The *gpd*A promoter, the iGFUGT fragment, and reversed *gpd* promoter were inserted into the pAN7-1 plasmid which was cut by *Bam*HI and *Hin*dIII using the ClonExpress® II One Step Cloning Kit (Vazyme, Nanjing, China) to inhibit the expression of *GFUGT* by RNA interference (RNAi), and the RNAi plasmid was named pAN7-iUGT. The OEGFUGT fragment was amplified by the primers OEGFUGT-F/R (shown in Supplemental Table S1) for overexpression vector construction. The overexpression vector pAN7-OEUGT was reconstituted with the *gpd*A promoter, and the OEGFUGT fragment was inserted into pAN7-1 plasmid which was cut by *Bam*HI and *Hin*dIII, using the ClonExpress® MultiS One Step Cloning Kit (Vazyme, Nanjing, China). All primers used in this study and their functions were listed in Supplementary Table S1.

Protoplasts were prepared using enzymatic hydrolysis as previously described (Cui et al. 2019). Briefly, mycelium was cultured for 7 days and digested using 2% lywallzyme (Guangdong Institute of Microbiology, Guangdong, China) at a rate of 1 mL of enzyme solution per 100 mg of mycelium. The plasmid pAN7-iUGT and pAN-OEUGT were transformed into protoplasts using PEG-6000 with pAN7-1 as a control strain (Sato et al. 2014). The transformed protoplasts were solubilized in the upper precooling MYG medium and spread on the lower MYG selection medium containing 100 μg/mL hygromycin B. The concentration of hygromycin B was pre-tested referring to two previous studies (Jiang et al. 2022; Zan et al. 2020a). Protoplasts were incubated in a constant temperature incubator at 28 °C for 10 days. The positive transformants were verified by PCR amplifying a fragment containing the partial *gpd*A promoter and the hygromycin phosphotransferase (*hph*) gene fragment with primer pair-HPH-F/R and subsequent gel electrophoresis. Transformants with clear amplification bands were selected, and the transcript levels of *UGT88A1* were analyzed by qRT-PCR for further selection. The control strain with empty plasmid, the overexpression strain, and the silenced strain of *G. frondosa* were named CK, OEUGTn, and iUGTn, respectively.

### Determination of fermentation performance and purification of polysaccharides

Determination of fermentation performance and purification of EPS referred to a previous method (Wang et al. 2021). Briefly, the supernatant and mycelium were separated by centrifugation after 7 days of fermentation. The mycelium was washed with distilled water and dried to a constant weight at 60 °C to obtain the biomass. The supernatant was mixed with ethanol to 80% and kept at 4 °C for 24 h. The precipitate was collected by centrifugation and freeze-dried to collect crude EPS. EPS production was determined by the phenol–sulfuric acid method (Dubois et al. 1956). Crude EPS was redissolved in distilled water and treated with a TOYOPEARL DEAE-650 cellulose anion exchange column (TOSOH Co, Ltd., Tokyo, Japan). The neutral and acidic components were separated under the elution of distilled water and 0.2 mol/L NaCl solution, at a flow rate of 3 mL/min, respectively, and processed with a Sephadex G-100 gel chromatographic column (Pharmacia Corporation, Stockholm, Sweden). The obtained polysaccharide solutions were subjected to rotary evaporation and freeze-dying to collect purified EPS.

### Mycelium morphology observation and pressure sensitivity test

The mycelium with a diameter of 5 mm was removed from PDA plates of control, overexpression, and silenced strains, respectively, using a puncher, and inoculated onto new plates. A sterile coverslip was inserted angularly into the plates and removed when the mycelium grew to two-thirds of the coverslip. The mycelium was fixed with 10% formalin for 1 h, air-dried, and stained with 1% (M/V) Congo red solution for 12 h. Then, the coverslip was inversely transferred onto a slide with a drop of 100 μl PBS buffer and placed under a microscope (Olympus, Tokyo, Japan) for observation. As described above, mycelia were removed from PDA plates of different transformants using a puncher, respectively, and inoculated on PDA plates containing 0.1 M NaCl, 0.2 mM farnesol, 5 mM hydrogen peroxide, and pH 9. The colonies were observed after 15 days of incubation at 28 °C.

### Chemical analysis and structural characterization of EPS

Each EPS solution (1 mg/mL) was scanned at 200–400 nm using a BioPhotometer D30 (Eppendorf, Hamburg, Germany). The EPS samples were analyzed using a VERTOR22 Fourier transform infrared spectrometer (Bruker Corporation, Billerica, MA, USA). The dried polysaccharides samples were mixed with KBr, then grinded and pressed into thin slices for FT-IR (Fourier transform infrared spectrometry) at wavelengths ranging from 4000 to 400 cm^−1^ (Shen et al. 2018). The monosaccharide composition of polysaccharides was analyzed by referring to the method used by Shen et al. (2018). The morphology of EPS samples was observed using a scanning electron microscope (SEM) (FEI Co, Ltd., Hillsboro, OR, USA). The samples were fixed on conductive and gold films formed by ETD-800 ion sputter (Elaborate Technology Development Ltd., Beijing, China). The polysaccharide samples were observed at a voltage of 5.0 kV with the scanning electron microscope (FEI Co, Ltd., Hillsboro, OR, USA).

### Antioxidant activity assay

The EPS samples of control and overexpression strain were diluted with distilled water into different concentrations of 0.5, 1.0, 1.5, 2.0, and 2.5 mg/mL. Antioxidant activity was evaluated by scavenging the activity of 1,1-diphenyl-2-picrylhydrazyl radical (DPPH), 2,2′-azino-bis (3-ethylbenzothiazoline-6-sulphonic acid) (ABTS radical), and hydroxyl radicals referring to the method used in a previous study (Shen et al. 2018).

### Transcriptional analysis by qRT-PCR

The transcriptional levels of cell wall integrity (CWI) pathway Rho1 (OBZ75295.1), mitogen-activated protein kinase (SLT2/MPK1, OBZ69932.1), serine/threonine protein kinase (Bck1/Slk1, OBZ67177.1), and polysaccharide synthesis–related gene glucan synthase (GLS, OBZ75851.1), phosphomannose isomerase (PMI, OBZ74466.1), mannose-1-phosphate gualyltransferase (GMP, OBZ67278. 1), UDP-xylose-4-epimerase (UXE, OBZ68549.1). and UDP-glucose epimerase (UGE, OBZ68550.1) were evaluated in control, overexpression and silenced strains by qRT-PCR with *GADPH* gene as an internal reference. The primer sequences used to amplify the target fragments are shown in Supplemental Table S1.

### Statistical analysis

Data were expressed as mean ± standard deviation (*n* = 3) of triplicates and analyzed by ANOVA or independent samples *t*-test using the SPSS 22.0 statistical software (Leech et al. 2014). Significance levels were set at *P*-value ≤ 0.05.

## Results

Cloning of the gene from *G. frondosa* and plasmid construction.

**Fig. 1:**
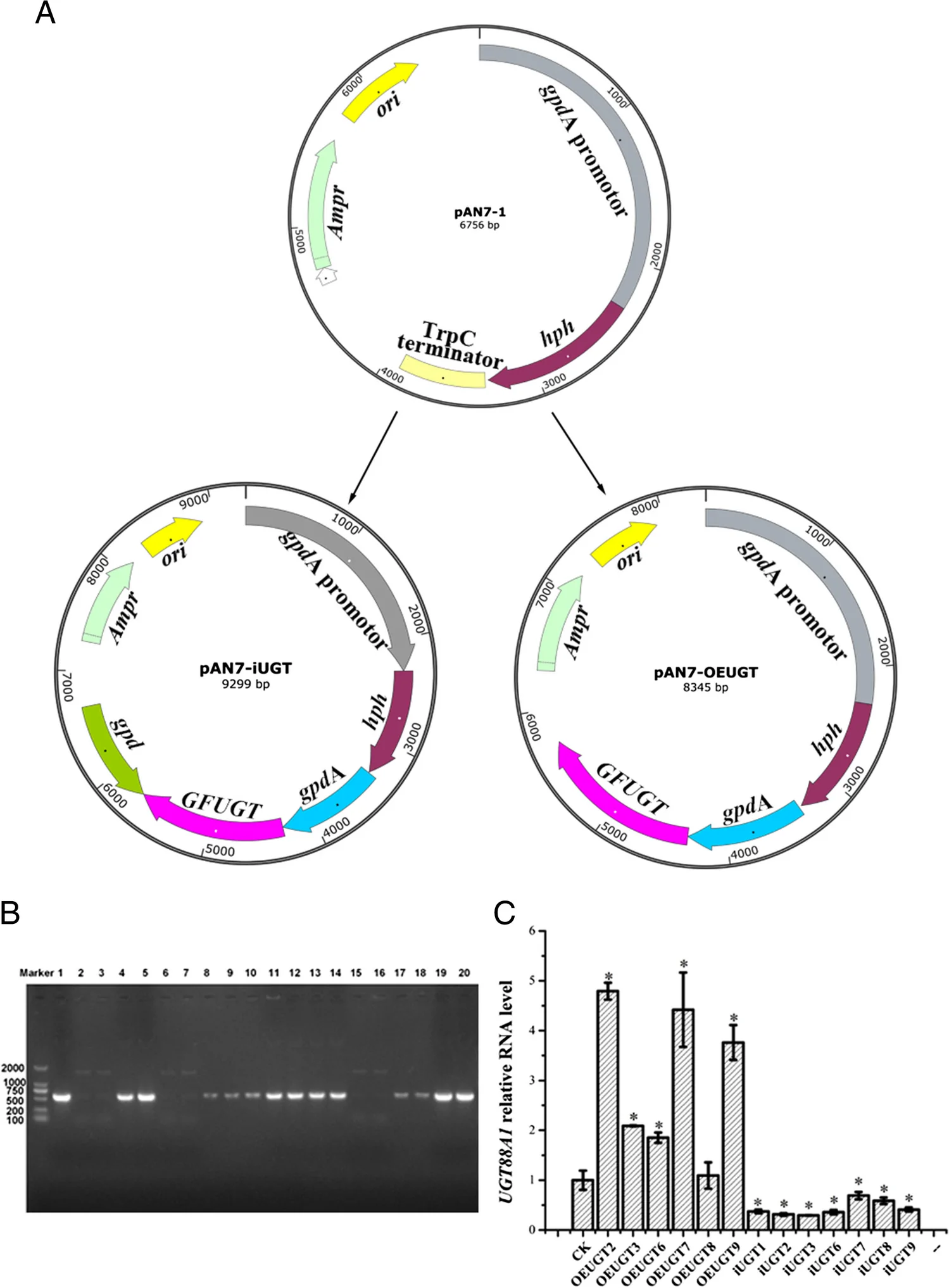
**A** The fragments GPDA-UGT and GPD-UGT-GPDA were ligated to the linearized vector to construct pAN7-OEUGT and pAN7-iUGT using pAN7-1 as the starting plasmid. **B** The genomic integrated resistance fragments of overexpression transformants and silenced transformants were verified using PCR and gel electrophoresis: lane 1: plasmid control (with *gpd*A promoter and revised *gpd* promoter), lane 2: control strain (CK), lanes 3–11: overexpression transformant (OEUGT1-9), and lanes 12–20: silenced transformant (iUGT1-9); bright bands indicate positive strains. **C** qRT-PCR and gel electrophoresis were used to analyze the transcriptional levels of UGT88A1 in the positive overexpressing and the silencing strains. Each strain was cultured in three 250 mL grooved Erlenmeyer flasks containing 100 mL of the fermentation medium for biological replicates. Then, the total RNA was extracted after 7 days of fermentation. All samples were performed in triplicates for technical replicates in qRT-PCR experiments. The data are expressed as means ± standard deviations (*n* = 3). The asterisk indicate a significant difference with CK (control strain) (*P* < 0.05, *n* = 3)

*Grifola frondosa* strain 9006–11 has an annotated complete genome sequence in the NCBI database under the accession number ASM168373v1. Based on this, we identified the *UGT88A1* coding sequence with a length of 1389 bp (Supplementary Fig. S1), which encodes a protein of 462 amino acids, that had a predicted molecular weight of 50.97 kDa and an isoelectric point (pI) of 5.29. To investigate the influence of *UGT88A1* on the growth and polysaccharide synthesis of *G. frondosa*, two promoters, *gpd*A and *gpd*, were used to construct the silencing and overexpression vectors, pAN7-iUGT and pAN7-OEUGT (Fig. 1A). The GFUGT2-F/R primer pair (Supplementary Table S1) was used to amplify the cassette encompassing the *gpd* (200 bp), *UGT88A1* (1389 bp), and *gpd*A (200 bp) sequences from the pAN7-iUGT vector. Subsequent sequencing followed by a BLAST search confirmed that the cloned sequence was identical to that found in the genome.

### Transformation and strain selection

The PEG 6000 protoplast transformation method (Cui et al. 2019) was used to introduce the two plasmids into the wild-type strain of *G. frondosa*. Specific primers (Supplemental Table S1) were used to amplify a 600-bp fragment spanning the *hph* resistance gene and promoter to confirm positive transformants. As shown in Fig. 1B, there were bright bands between the 500- and 700-bp DNA size markers in some silencing and overexpression samples, which were positive transformants. Positive transformants were selected based on transcription level, growth, and fermentation performance. To quantify the transcription level of *UGT88A1*, total RNA was extracted from positive transformants, followed by the generation of a cDNA library for qRT-PCR. As shown in Fig. 1C, the transcription levels of all overexpression strains were up-regulated compared to the control strain, whereas the expression of *UGT88A1* was significantly downregulated in the silenced strains. Subsequently, the OEUGT2 strain with the highest overexpression level (4.79-fold increase) and the iUGT3 strain with the strongest downregulation (0.30 relative expression level) were selected for further experiments.

### Effect of *UGT88A1* on the growth and fermentation performance of *G. frondosa*

**Fig. 2:**
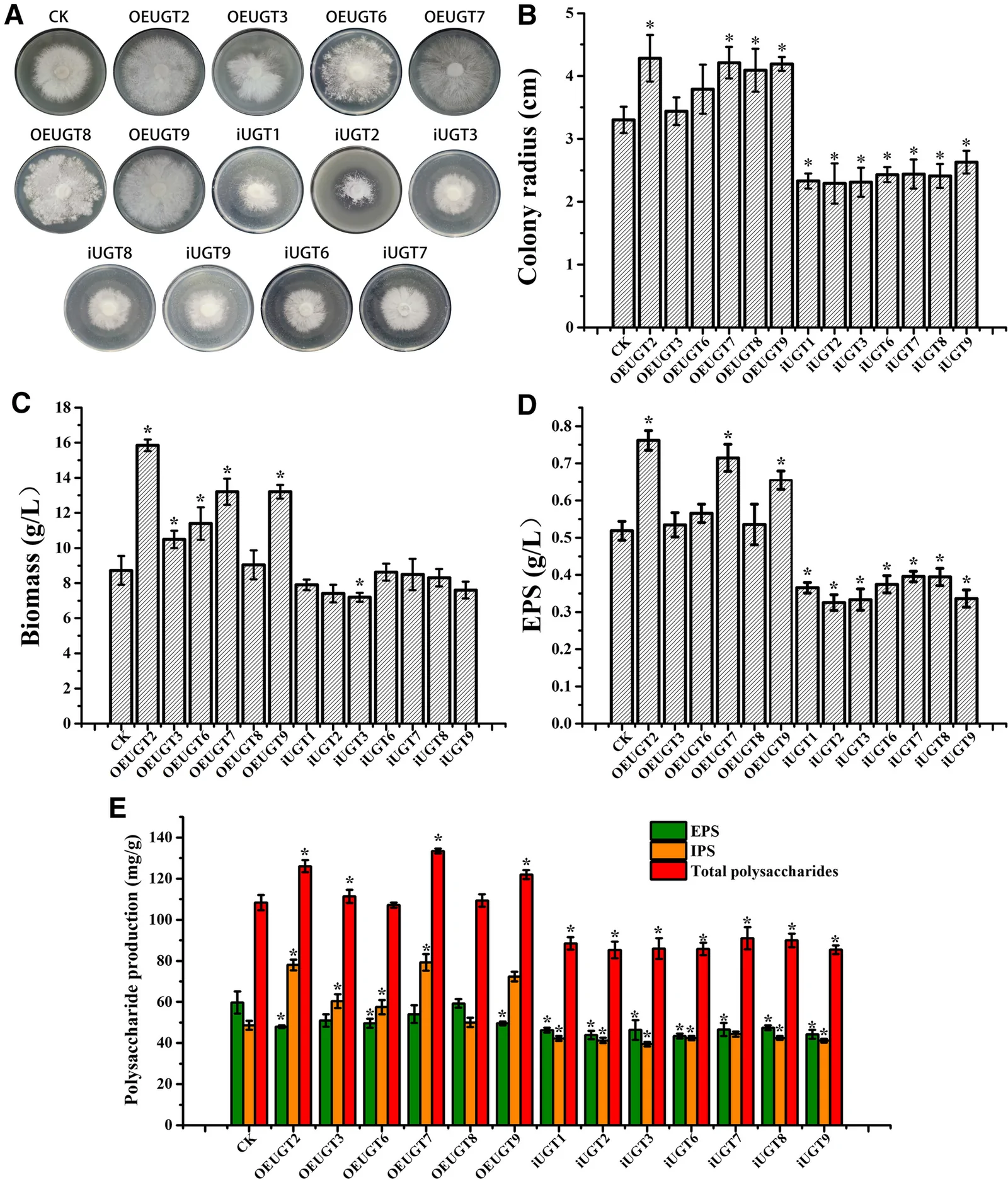
**A** CK, OEUGTn, and iUGTn strains (positive strain detected using PCR and gel electrophoresis) colony growth. **B** The colony radius of CK, OEUGTn, and iUGTn strains were each cultured on PDA medium, and taken the average of three measurements after growing for 15 days; Fermentation performance: biomass (**C**), extracellular polysaccharide production (**D**), and polysaccharide production per unit mycelium (**E**) of control (CK), overexpressing (OEUGTn), and silenced strains (iUGTn). Each strain was cultured in three 250 mL grooved Erlenmeyer flasks containing 100 mL of the fermentation medium for biological replicates. All samples were performed in triplicates for technical replicates in biomass, EPS production, and total polysaccharide production measurement. The data are expressed as means ± standard deviations (*n* = 3). The asterisk indicates a significant differences compared to the control strain (CK) (*P* < 0.05, *n* = 3)

To investigate the influence of the *UGT88A1* gene on the mycelial growth of *G. frondosa*, we observed and measured the growth of single colonies of all positive transformants selected using PCR and gel electrophoresis on potato dextrose agar (PDA) medium plates (Fig. 2A). As shown in Fig. 2B, the colony of the control strain reached a diameter of 3.30 ± 0.21 cm (*n* = 3) after 15 days of growth on the PDA plate. The colonies of most overexpression strains were larger than that of control strains; the OEUGT2 overexpression strain reached 4.28 ± 0.37 cm (*n* = 3). The colonies of silencing strains were smaller than those of the control strains, and the iUGT3 strain reached 2.29 ± 0.32 cm (*n* = 3). After 7 days of fermentation in 100-mL shake-flasks, the biomass of all overexpression strains was higher than that of control strains, and the strain OEUGT2 reached 15.85 g/L, representing an 81% increase over the control strain (8.73 g/L). In contrast, the iUGT3 strain reached a dry cell weight of 7.19 g/L, which was 18% lower than that of the control (Fig. 2C).

As shown in Fig. 2D, The EPS production by OEUGT2, OEUGT7, and OEUGT8 was significantly up-regulated compared to the control strain. The OEUGT2 strain accumulated 0.76 g/L of EPS, representing a 46% increase over the control strain (0.52 g/L). The EPS production of all the silenced strains decreased, and the iUGT3 strain only accumulated 0.33 g/L, which was 35% less than that of the control strain. IPS production increased in the overexpression strain, whereas that in the silencing strain decreased. OEUGT2 accumulated 78.01 mg/g of IPS, which was 60% higher than that in the control strain (48.63 mg/g), whereas the iUGT3 strain (39.54 mg/g) accumulated 19% fewer IPSs (Fig. 2E). For a more stringent comparison, the total polysaccharide production capacity of each strain was further quantified based on dry cell weight, as shown in Fig. 2E. OEUGT2 produced 126.05 mg/g total polysaccharides, which was 16% higher than that produced by the control strain (108.35 mg/g). The iUGT3 strain produced 85.96 mg/g of total polysaccharides, which was 20% lower than that produced by the control strain.

### Effect of *UGT88A1* on mycelial extension and cell wall stress in *G*. *frondosa*

**Fig. 3:**
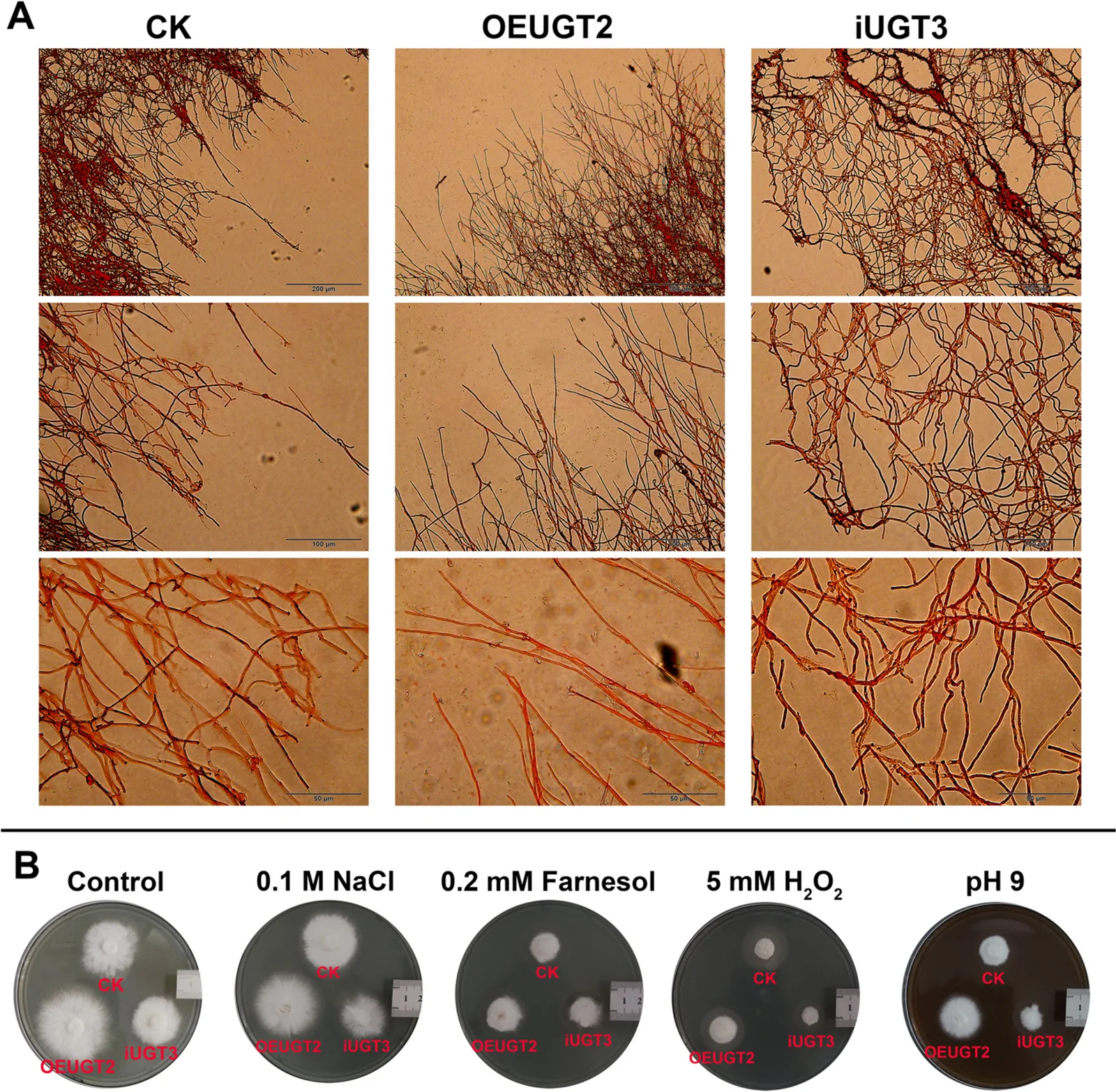
Morphological observation and cell wall pressure sensitivity analysis of *G. frondosa*. **A** Mycelial morphology observation of CK, OEUGT2, and iUGT3 strains growing on a PDA plate. The lengths of the solid lines in the figure are represented as 200 μm (10 × magnification), 100 μm (20 × magnification), and 50 μm (40 × magnification). **B** Cell wall pressure sensitivity test of CK, OEUGT2, and iUGT3 strains; the culture medium from left to right is PDA and PDA with 0.1 M NaCl, PDA with 0.2 mM farnesol, PDA with 5 mM H_2_O_2_, and PDA with pH 9

To further investigate the effect of *UGT88A1* on mycelial extension, the mycelial morphology of the colonies on agar plates was investigated (Fig. 3A). At 40 × magnification, the OEUGT2 strain exhibited less branched and longer hyphae than the control strain. In contrast, the iUGT3 strain exhibited a net-like mycelial pattern, with more branches and curled hyphae compared to the control strain. To assess the potential influence of *UGT88A1* on cell wall stress sensitivity in *G. frondosa*, the cell wall pressure resistance was measured. As shown in Fig. 3B, the iUGT3 strain exhibited increased sensitivity to the high osmotic pressure of the 0.1 M NaCl solution, while the OEUGT2 strain produced a larger colony diameter than the control. In alkaline medium (pH 9.0), the increased sensitivity of the silencing strain was more pronounced, whereas the overexpression strain was more resistant than the control based on the increased colony diameter. We also tested the potential influence of *UGT88A1* on oxidative stress resistance. Farnesol can affect oxidative stress in the mycelia (Wang et al. 2021). In the presence of 0.2 mM farnesol, no significant differences were observed in the growth of the three strains. However, all strains showed low tolerance to 5 mM H_2_O_2_, indicating a similar trend in stress sensitivity as observed for osmotic stress and high pH stress.

### Effect of *UGT88A1* on the properties, structure and antioxidant activity of EPSs in *G. frondosa*

The crude polysaccharides isolated from the control, overexpression, and silenced strains were separated into neutral polysaccharide (NP) and acidic polysaccharide (AP) fractions and named CK-NP, CK-AP, OE-NP, OE-AP, iUGT-NP, and iUGT-AP.

#### UV absorption spectra of the EPSs

**Fig. 4:**
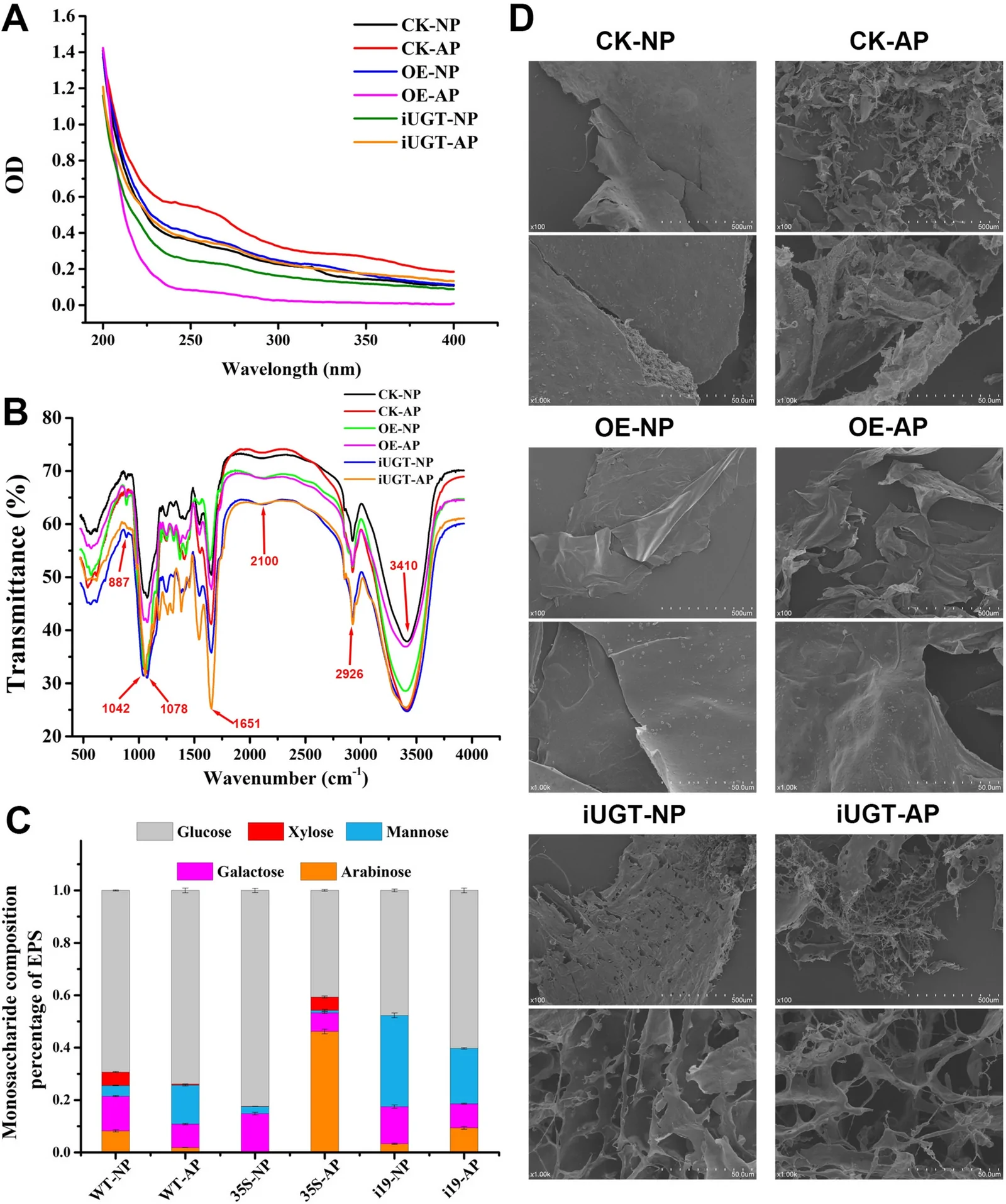
UV scanning (**A**), FT-IR spectral scanning (**B**), monosaccharide composition analysis (**C**), and SEM image (**D**) of CK-NP, CK-AP, OE-NP, OE-AP, iUGT-NP, and iUGT-AP. Each EPS sample was performed in triplicates for technical replicates in the measurement of monosaccharide composition. The data are expressed as means ± standard deviations (*n* = 3). The asterisk indicates a significant difference compared to the control strain (CK) (*P* < 0.05, *n* = 3)

None of the polysaccharide fractions showed detectable absorption peaks at 260 or 280 nm (Fig. 4A), indicating no significant contamination with nucleic acids or proteins. Thus, the polysaccharide samples were of sufficiently high purity for further analysis.

#### Fourier-transform infrared (FT-IR) spectra of the EPSs

FT-IR spectroscopy was used to determine the chemical composition of the EPSs of *G. frondosa.* As shown in Fig. 4B, all polysaccharide fractions exhibited similar infrared spectra. The strong peaks at 3410 and 2926 cm^−1^ were attributed to the -OH and C-H stretching vibrations of the sugar moieties, respectively (Han et al. 2014; Yang et al. 2006). The absorption peak at 1651 cm^−1^ was attributed to the presence of carboxyl groups, as observed for uronic acids. The peaks at 1200–1400 cm^−1^ were attributed to C-H bending vibrations, while those in the range of 1100–1010 cm^−1^ indicated that the polysaccharides contained furanose rings. Galactose and glucose have the strongest absorption peaks around 1078 and 1035 cm^−1^, respectively (Kacurakova et al. 2000). Accordingly, the peaks at 1078 and 1042 cm^−1^ indicated that all polysaccharide fractions contained glucose and galactose, respectively. Finally, the peak at 887 cm^−1^ indicated that all EPS fractions of the three strains of *G. frondosa* contained β-glycosidic bonds (Han et al. 2014).

#### Monosaccharide composition of the polysaccharides

Former studies reported that the EPSs of *G. frondosa* had antioxidant activity and anti-tumor properties closely related to their monosaccharide composition (Chimilovski et al. 2011; Shen et al. 2018; Zhang et al. 2016). Accordingly, it was pertinent to investigate the monosaccharide composition of EPSs isolated from the three strains of *G. frondosa* in this study. As shown in Fig. 4C, the CK-NP, CK-AP, and OE-AP fractions contained glucose, mannose, galactose, xylose, and arabinose. However, arabinose was not detected in the OE-NP fraction, whereas the two fractions isolated from the silenced strains (iUGT-NP and iUGT-AP) did not contain any detectable levels of xylose. The most abundant monosaccharide in CK-NP, CK-AP, OE-NP, iUGT-NP, and iUGT-AP was glucose, accounting for 47.69–82% of the total. However, the arabinose content in OE-AP exceeded that of glucose (46.16 vs. 40.70%). The relative abundance of mannose in the silenced strain was increased compared to that in the control (21.07–34.82%), whereas it was significantly decreased in the overexpression strain (0.97–2.63%). Thus, the transcriptional level of *UGT88A1* had a major influence on the monosaccharide composition of the EPSs of *G. frondosa*.

#### Scanning electron microscopy (SEM) analysis of the ultrastructure of polysaccharides

SEM was used to investigate the ultrastructure and morphology of polysaccharides from the three strains. As shown in Fig. 4D, CK-NP, OE-NP, and iUGT-NP fractions containing neutral polysaccharides exhibited an irregular layered structure. However, the surfaces of CK-NP and OE-NP were smooth, whereas those of iUGT-NPs exhibited numerous pores. Among the acidic polysaccharides, CK-AP and OE-AP exhibited layered structures with smooth surfaces, whereas CK-AP contained scattered particles. In contrast, the ultrastructure of iUGT-AP was significantly different, with a net-like appearance, fibrous structure, and a smooth surface at high magnification. Thus, the transcription level of *UGT88A1* also significantly influenced on the ultrastructural morphology of polysaccharides from *G. frondosa*.

#### Antioxidant activity of polysaccharides from G. frondosa

**Fig. 5:**
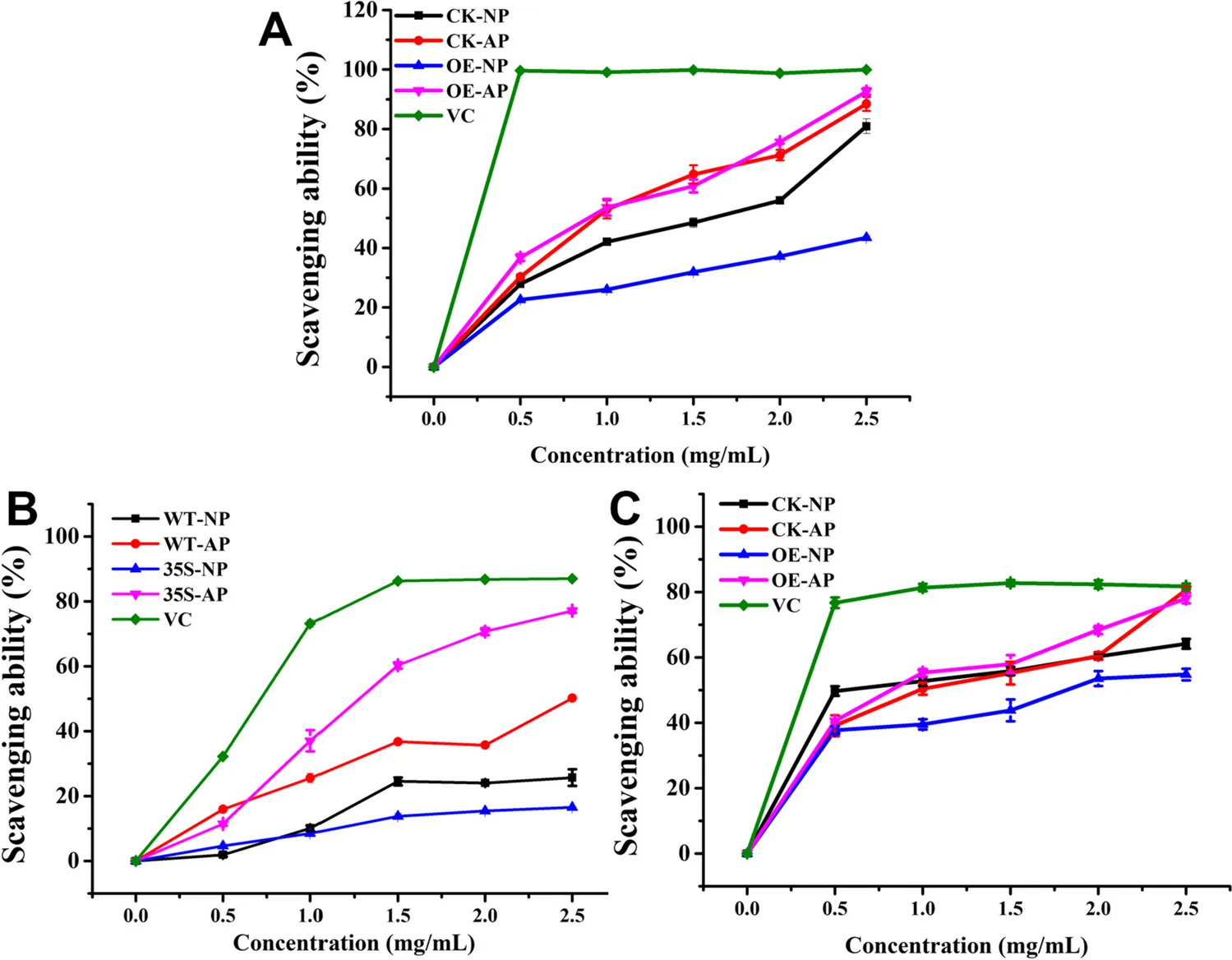
Antioxidant activity of CK-NP, CK-AP, OE-NP, and OE-AP. **A** ABTS^+^ radical scavenging activity. **B** Hydroxyl radical scavenging activity. **C** DPPH radical scavenging activity. All experiments were conducted in a 96-well plate, with three reaction wells set for each experiment as technical replicates. The data are expressed as means ± standard deviations (*n* = 3). The asterisk indicates a significant differences compared to the control strain (CK) (*P* < 0.05, *n* = 3)

In this study, the antioxidant activities of the CK-NP, CK-AP, OE-NP, and OE-AP polysaccharide fractions were assessed using ascorbic acid (vitamin C, VC) as a positive control in ABTS radical, hydroxyl, and DPPH radical scavenging assays. As shown in Fig. 5A, there was a rapid increase in ABTS radical scavenging ability when the polysaccharide concentration was increased from 0 to 0.5 g/L, but it was lower than that of VC. With a further increase in polysaccharide dosage, the ABTS radical scavenging activity of OE-NP reached saturation. At a polysaccharide concentration of 2.5 g/L, the ABTS radical scavenging activities of CK-NP, CK-AP, OE-NP, and OE-AP were 81, 88, 43, and 93%, respectively. These results confirm that the acidic polysaccharides of *G. frondosa* have stronger ABTS radical scavenging activity than neutral polysaccharides, which is consistent with the result of a study by Wang et al. (2021).

Similarly, the hydroxyl radical scavenging ability increased with the dose of polysaccharides (Fig. 5B) but was lower than that of VC. At a dose of 2.5 g/L, the hydroxyl radical scavenging activities of CK-NP, CK-AP, OE-NP, and OE-AP were 26, 50, 17, and 77%, respectively. As shown in Fig. 5C, the DPPH radical scavenging ability of all polysaccharide fractions increased in a dose-dependent manner but was lower than that of VC. The DPPH radical scavenging activities of CK-NP, CK-AP, OE-NP, and OE-AP reached 64, 81, 55, and 78%, respectively, which were lower than the 81.73% radical scavenging activity of VC.

### Effect of *UGT88A1* on the mRNA expression of genes related to cell wall integrity and polysaccharide synthesis

**Fig. 6:**
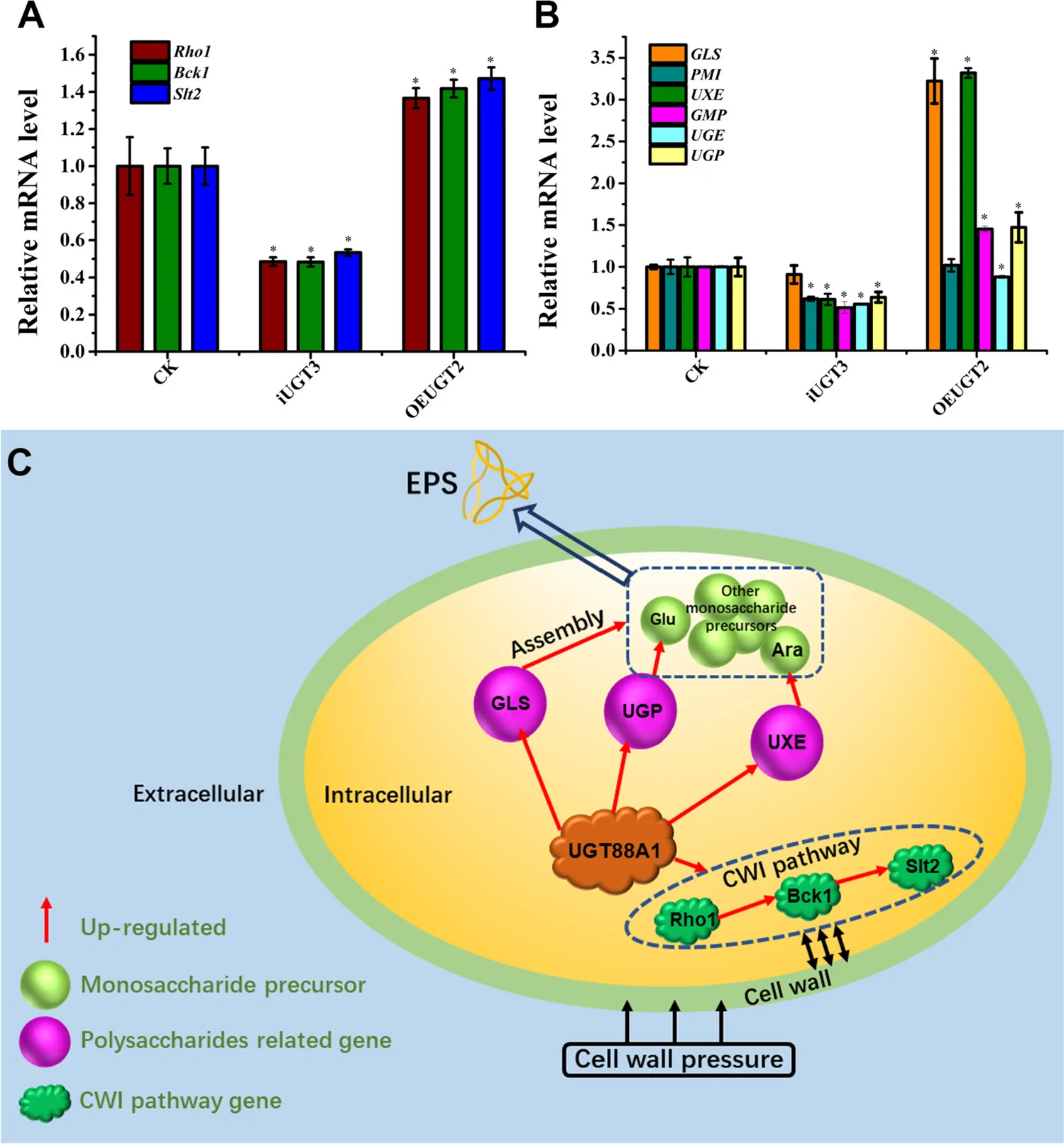
qRT-PCR analysis of mRNA expression of genes involved in cell wall integrity pathway (*Rho1*, *Bck1*, and *Slt2*) and polysaccharide synthesis (*GLS*, *PMI*, *UXE*, *GMP*, *UGE*, and *UGP*) of CK, OEUGT2, and iUGT3 after 7 days of submerged cultivation. **A** Transcription level analysis of CWI pathway genes. **B** Transcription level analysis of genes related to polysaccharide synthesis. Each strain was cultured in three 250 mL grooved Erlenmeyer flasks containing 100 mL of the fermentation medium for biological replicates. Then, the total RNA was extracted after 7 days of fermentation. All samples were performed in triplicates for technical replicates in qRT-PCR experiments. **C** Schematic diagram of the effect of *UGT88A1* on genes related to two pathways in overexpression strain: The upregulation of *UGT88A1* transcription level led to the upregulation of CWI pathway gene transcription levels, and when mycelium was subjected to external pressure stress, the cell wall sensitivity decreased; *UGT88A1* affected the transcription level of *GLS*, *UGP*, and *UXE*, thereby promoting the synthesis of polysaccharides and the increase in arabinose. Samples of qRT-PCR analysis were performed in triplicates. The data are expressed as means ± standard deviations (*n* = 3). The asterisk indicates a significant differences compared to the control strain (CK) (*P* < 0.05, *n* = 3)

Because the transcriptional level of *UGT88A1* affects the growth and cell wall pressure sensitivity of *G. frondosa*, the mRNA expression levels of *Rho1, Bck1*, and *Slt2*, three crucial genes related to cell wall integrity, were also investigated (Dichtl et al. 2012; Zhang et al. 2018). As shown in Fig. 6A, *UGT88A1* overexpression upregulated the mRNA expression of *Rho1*. Similarly, the mRNA expression of *Bck1* and *Slt2* increased by 41% and 47%, respectively. Conversely, RNAi silencing of *UGT88A1* downregulated the mRNA expression of all three genes (Fig. 6C).

As shown in Fig. 6B, the genes investigated were *GLS*, *UGP*, *PMI*, *GMP*, *UGE*, and *UXE*. GLS is the key enzyme involved in glucan synthesis. UGP, PMI, GMP, UGE, and UXE participate in the synthesis of different monosaccharide precursors in polysaccharide synthesis. UGP catalyzes the reversible interconversion of Glc-1-phosphate and UDP-Glc; PMI catalyzes the reaction that generates Fru-6-phosphate, the precursor of GDP-Man in polysaccharide synthesis; GMP catalyzes the synthesis of GDP-Man; UGE catalyzes the synthesis of UDP-Gal; and UXE catalyzes the synthesis of UDP-Ara (Wang et al. 2017). In the *UGT88A1* RNAi silencing strain, almost all investigated enzymes related to the supply of monosaccharide precursors for polysaccharide synthesis were downregulated. However, *GLS* was not induced with a significant change at the transcriptional level. In contrast, the expression of *GLS*, a key enzyme in monosaccharide assembly during polysaccharide synthesis (Cui et al. 2019), was upregulated 3.22-times in the *UGT88A1* overexpression strain. *UXE* is the key enzyme in UDP-arabinose synthesis (Wang et al. 2017), and its expression was increased 3.32 times in the *UGT88A1* overexpression strain. There was also a 47% increase in the expression of *UGP*, which is responsible for the synthesis of UDP-glucose.

## Discussion

Compared to other model filamentous fungi, genetic engineering methods have not been well developed for mushroom basidiomycetes. The construction of overexpression strains and RNA-mediated gene silencing are effective methods for identifying gene functions (Mu et al. 2012; Zan et al. 2020b). In this study, the role of the *UGT88A1* gene in *G. frondosa* was investigated by overexpression and RNAi silencing. It was found that the overexpression strains showed a significant increase in the transcript levels of *UGT88A1*, mycelial growth, and colony diameter of biomass, and production of EPSs and IPSs increased. The silenced strains showed the opposite trend. Overexpression of the *UGT88A1* gene mainly increased the biomass and intracellular accumulation of polysaccharides. In addition, the accumulation of EPSs during fermentation is related to the biomass. Although RNAi silencing of *UGT88A1* had a significant effect on biomass accumulation and polysaccharide synthesis, the effect was weaker than that of its overexpression. This finding agrees with recent reports that polysaccharide synthesis is related to mycelial growth in *G. frondosa* (Cui et al. 2019; Zan et al. 2020a).

A previous study on glucan synthase (*GLS*) indicated that *GLS* silencing inhibits the production of polysaccharides and decreases mycelial growth and colony extension (Cui et al. 2019). Therefore, mycelial morphology and pressure sensitivity tests were performed on the overexpressing and silenced strains. *UGT88A1* overexpression increases intracellular polysaccharide synthesis. As components of the fungal cell wall, IPSs directly influence cell-wall synthesis and mycelial growth (Krylov and Nifantiev 2019; Sakamoto et al. 2018). Therefore, it stands to reason that *UGT88A1* overexpression increased the synthesis of intracellular polysaccharides, which in turn promoted cell wall synthesis and mycelial extension. Cell wall pressure sensitivity testing offers a direct measure of cell wall integrity (Dichtl et al. 2012). In our study, by comparing the colony size under different cell wall and oxidative stress conditions, we found that the overexpressing strain had lower pressure sensitivity than the control strain, while the silencing strain had higher sensitivity. These results indicated that the transcriptional level of the *UGT88A1* gene significantly affected the oxidative stress sensitivity of *G. frondosa*, which may be related to the composition of the cell wall, as observed in *Aspergillus fumigatus* (Zhang et al. 2018). Glycosyltransferases are directly involved in the synthesis of cell wall polysaccharides by catalyzing the formation of glycosidic bonds (Scheible and Pauly 2004; Zabotina et al. 2021). Accordingly, the decrease in cell wall pressure resistance by *UGT88A1* silencing can be attributed to weakened cell wall integrity owing to the reduction in polysaccharide content. A recent study reported that the cell-wall integrity of *G. frondosa* is related to cell-wall formation and polysaccharide synthesis (Zan et al. 2020b). Cell-wall integrity is determined by cell-wall synthesis and remodeling, metabolic pathways, and external stress factors outside the cell. qRT-PCR results showed that *UGT88A1* overexpression upregulated the mRNA expression of *Rho1, Bck1*, and *Slt2*. Conversely, RNAi silencing of *UGT88A1* downregulated the mRNA expression of all three genes. Zan et al. (2020b) showed that *Rho1* gene silencing decreased the pressure sensitivity of the cell wall in *G. frondosa* and increased the rate of mycelial growth, which is in agreement with the result of our study here. They also demonstrated that the strain of silenced *UGP* in *G. frondosa* had thickened hyphae than those in the wild type, and the diameters of silenced strains were decreased compared with the wild type (Zan et al. 2020a). Similarly, in the present study, the scanning electron microscope showed the silenced strain exhibited a net-like mycelial pattern, with more branches and curled mycelia than the control strain. *Bck1* and *Slt2* encode mitogen-activated protein kinases related to cell wall integrity, which play important roles in promoting cell polarization and regulating osmotic pressure (Ma et al. 2020). Overall, our results indicated that *UGT88A1* overexpression increased polysaccharide production, which in turn promoted cell wall synthesis and reduced mycelium stress sensitivity. However, the underlying mechanism requires further investigation.

Although overexpression of *UGT88A1* increased the production of polysaccharides, it was unclear whether it had an effect on the functional properties. In vitro assays offer a simple method for assessing the antioxidant activities of polysaccharides. The antioxidant activities of the four tested polysaccharide fractions from different strains showed distinct differences. The results clearly showed that OE-AP had the highest hydroxyl radical scavenging ability, and OE-NP had the weakest, which may be related to more efficient Fe^2+^ chelation (Fan et al. 2012). It has been reported that an increased proportion of uronic acid in polysaccharides increases their chelating ability (Yarley et al. 2021). Therefore, further studies are needed to determine whether the uronic acid content in OE-AP is higher than that in the polysaccharides from the control strain. Monosaccharide composition is considered one of the most important factors affecting the antioxidant capacity of polysaccharides (Su and Li 2019). Accordingly, the differences in antioxidant activity may be related to differences in monomer composition among the polysaccharides investigated in this study. It has been reported that the PKP-E-2–1 polysaccharide extracted from the pine cones of *Pinus koraiensis* had a higher arabinose content and antioxidant activity than other polysaccharide fractions (Hua et al. 2020). Another study reported that the EPS (mainly L-Ara) obtained from the fermentation of *G. frondosa* JSULY2020 strains had significant antioxidant activity (Yang et al. 2021). Similarly, OE-AP had the highest arabinose content in this study, which may explain its high antioxidant activity. However, in a previous study on polysaccharides of four common edible mushrooms, it was found that the content of L-Ara was not the key factor affecting the antioxidant activity of the polysaccharides (He et al. 2012). Because of the complex relationship between the biological activity of natural polysaccharides and their molecular weight, monosaccharide composition, glycosidic bonds, and other chemical properties, the mechanism of biological activity found in polysaccharides of *G. frondosa* and its relationships with structure remained uncertain; therefore, further research is needed to focus on the structure and function of *G. frondosa* polysaccharides.

UV absorption spectroscopy, FT-IR spectroscopy, monosaccharide composition measurements, and SEM analysis are common methods for detecting the physical and chemical properties of polysaccharides. The results showed that overexpression and silencing of *UGT88A1* affected the ultrastructural morphology and monosaccharide composition of different *G. frondosa* polysaccharide components. Fungal polysaccharide synthesis is catalyzed by a large number of diverse enzymes, and we decided to investigate the mRNA expression of a subset of crucial enzymes related to polysaccharide synthesis in strains with different *UGT88A1* transcript levels (Wang et al. 2017). The results showed that the mRNA levels of all the investigated enzymes related to the supply of monosaccharide precursors for polysaccharide synthesis were downregulated in the silenced strain, but *GLS* did not exhibit a significant change. Accordingly, the observed weakening of polysaccharide synthesis following *UGT88A1* silencing may be due to the reduced synthesis of activated monosaccharide precursors. A recent study has demonstrated that the product of *UGT88A1* of *G. frondosa* has a substrate preference for oligosaccharide with a comparatively high degree of polymerization (≥ 6) when using UDP-glucose as a donor in vitro, and extended glucan chains (Yyl et al. 2022). Here, we found that *UGT88A1* overexpression upregulated the mRNA expression of *GLS*, which promoted the assembly and polymerization of polysaccharides. This could also explain the increased polysaccharide production observed in the overexpression strain. The mRNA levels of *UXE* and *UGP* were also upregulated in the overexpressing strain. In the polysaccharide synthesis pathway of *Ganoderma lucidum*, UDP-glucose is a precursor for UDP-arabinose synthesis (Lin et al. 2016). Accordingly, our results indicated that increased levels of UDP-glucose may have, in turn, increased the synthesis of UDP-arabinose. However, the role of UDP-Ara in cell wall production and strain growth in fungi has not yet been reported. In a study on *A. thaliana*, L-Ara was found to be a key component of cell wall polymers, glycoproteins, flavonoids, and signaling peptides, and the biosynthesis of UDP-Ara mainly occurred through the epimerization of UDP-Xyl by UXE in the Golgi lumen (Rautengarten et al. 2017). Therefore, high expression of UXE in the overexpression strain may promote the synthesis of UDP-Ara and the growth of the strain. These results confirmed that UGT88A1 influenced multiple enzymes responsible for the synthesis of activated monosaccharide precursors to different extents, which may have directly affected the monosaccharides profile of the investigated polysaccharides. Future studies should address whether a synergistic relationship exists.

## Supplementary Information

Below is the link to the electronic supplementary material.

Supplementary file1 (PDF 598 KB)

## Data Availability

All data generated or analyzed during this study are included in this published article (and its supplementary information files).
