# Supplementary material for: A newly discovered glycosyltransferase gene UGT88A1 affects growth and polysaccharide synthesis of Grifola frondosa
Source: Appl Microbiol Biotechnol. 2024 Feb 29;108:246. doi: 10.1007/s00253-024-13062-0 (PMC10904514; doi:10.1007/s00253-024-13062-0)
Supplement: Supplementary file 1 [file 253_2024_13062_MOESM1_ESM.pdf]

Applied Microbiology and Biotechnology

Supplementary Material

A newly discovered glycosyltransferase gene *UGT88A1* affects growth and polysaccharide synthesis of *Grifola frondosa*

Jian Li, Bao-xin Wang, Jie Zhang, Na Han, Shu-ting Liu, Wen-ji Geng, Shi-ru Jia, Yan-ru Li, Quan Gan, Pei-pei Han*

State Key Laboratory of Food Nutrition and Safety, Key Laboratory of Industrial Fermentation Microbiology, Ministry of Education, College of Biotechnology, Tianjin University of Science and Technology, Tianjin 300457, P.R. China

* Corresponding author: State Key Laboratory of Food Nutrition and Safety, Key Laboratory of Industrial Fermentation Microbiology, Ministry of Education, College of Biotechnology, Tianjin University of Science and Technology, Tianjin 300457, P.R. China. Email: pphan@tust.edu.cn; Tel: +86 22 60601598; Fax: +86 22 60602298

1	ATGGTCAGGC	TGCGGGTGAT	TGTGGTCACC	TTCCTCACGA	CGGCCGCACT	TTACGATCGC
61	ATCAAGTCTG	AGATATCGCG	AAGCTTTGAG	CCAAACGAAG	AGCATCTCCT	CGATCGCATT
121	AGGGTGATCG	CGCTGCAGAA	GAACCTTCAA	GACCACTTTG	GCGGAGACAC	TCTTGACACT
181	GCATTTGAAG	ACGCATACAG	CAAGCTCATT	TCGGAGGAGC	CACTGACATG	CGTGAAGACC
241	GGTACTTCGT	ACGACTCCGT	TCGCAGCCCT	GATGCAGCCA	TCGTGGACTT	TTTCGCTATC
301	GTTCCATTCA	ATGCGGCCAA	AAAACTCAAC	AGAAATGGCG	AGGAGAGAAG	GAACCTCCGT
361	GCGAAAGTGG	AGGCCGAGGT	CAAACGGTCC	GGAAGACCTT	TCAATTTGGT	CGCCACGGAG
421	GTTCTCTTTG	GCAGTGATGG	ATCGGTCGTC	CGCATTCCTG	GACTGCCGCC	TATGTATGAC
481	CACGAATACA	ACCCGCAAGA	CTTCACGTTC	TCCGAAGATC	TCGGTGCGAA	GATACTGCTT
541	ATGACCTACG	ACACGCTCGA	AGCATCCGAT	GGAGTCCCTC	TGCTTAGCGC	GGAGGCGTAC
601	GAACCAGAAT	GCGTCGCCGC	CGTCAGGGAA	TGGTTCGCGC	AGACCTCCCG	CAGCGTCACC
661	GTTTGCGGAC	CGCTGCTGCC	CTCTGGGAAG	AATGCTGCCG	CTCACGAGAA	GCAGCAGTCG
721	AGCGAAGGCA	ACGAAATCCA	GAAGTTCCTG	GACTCGACCC	TCGAGTCCCA	CGGAACGCAT
781	TCGCTGCTTT	ATATATCGTT	CGGATCTATG	TTCTGGCCTG	CAAAGCCGGA	TGTCATATGG
841	GCTTTCCTTG	ACGTTGTCAT	GGAGCTAAAG	ATCCCCTTCA	TCTTGAGCCA	CGCATCGCCA
901	TTGGCCGTTG	TACCTGAAGA	GGTTTCCCAG	AAGGTCCGCG	AGTATGGACT	TGGTTTACTC
961	TCACCATGGA	CCCCACAGCA	GACGATTCTC	AACCACCCTG	CGACGGGCTG	GTTCGTAGCC
1021	CACGGCGGCC	ACAACGGCGT	GCTAGAGGCA	ACAACCGCAG	GCGTGCCACA	AATATTCTGG
1081	CCCTTCGCCG	CGGACCAGCC	ACTCAATGCC	GTGCACCTCA	CGCACAACCT	CGACGTCGCG
1141	TACGAGCTCA	TCGAGGTGCG	CACTGGCAAG	GGGCTCTTGC	CGATCCTGCG	TACTGGCTAC
1201	ACACCTGTAG	GCACGCCAGA	GGCCGTGCGG	AACGAAGCCA	AGGATGTGCT	CACGAAGGCA
1261	TTCGGCGAGG	ACGGAGAGCG	CAAGCGTGCG	AAGCTGCAGG	CGCTGAAAAA	GGCCCTCGCG
1321	GAGTCGTGGG	CGGAAGATGG	CCCTTCCAGG	CGTGATGTCG	AAGCGCTCCT	TGATTCTGTC
1381	GGTGTTTGA					

Figure S1 CDS (coding sequence) of *UGT88A1* from *G. frondosa*

Table S1 Primers used in the present study.

Primer	Sequence (5' to 3')	Description
GFUGT-F	ATGGTCAGGCTGCGGGTGAT	Cloning *GFUGT88A1* from *G. frondosa* cDNA library
GFUGT-R	TCAAACACCGACAGAATCAAG	
OEGFUGT-F	AGTCCCTGGTAGGCAGCTTTATGGTCAGGCTGCGGGTGATT	Construction of *GFUGT88A1* overexpression plasmid with fragment
OEGFUGT-F	CGACGGCCAGTGCCAAGCTTTCAAACACCGACAGAATCAAG	
GPDA-F	AGATGCCGACCGCGGGATCCGATTTCGGCACGGCTACGGAA	Construction of *GFUGT88A1* overexpression plasmid with *gpd*A promotor
GPDA-R	**ATCACCCGCAGCCTGACCATAAAGCTGCCTACCAGGGACTG**	
**iGFUGT-F**	**ACCCACCAGTATGGTCAGGCTGCGGGTGATTG**	Construction of *GFUGT88A1* silencing plasmid
**iGFUGT-R**	**AGGCAGCTTTTCAAACACCGACAGAATCAAGGA**	
**iGPD-F**	**GGCAAAGGAATAGAGTAGATGCCGACCGCGGGATCCGTTCG**	Construction of *GFUGT88A1* silencing plasmid with *gpd* promotor
**iGPD-R**	**GCCTGACCATACTGGTGGGTACAAATGACGTC**	
**iGPDA-F**	**CGGTGTTTGAAAAGCTGCCTACCAGGGACTGA**	Construction of *GFUGT88A1* silencing plasmid with *gpd*A promotor
**iGPDA-R**	**GTTGTAAAACGACGGCCAGTGCCAAGCTGATTTCGGCACGGC**	
**HPH-F**	**CGTATATGCTCCGCATTGGTCT**	PCR verification for anti-hygromycin positive transformants
**HPH-R**	**GGCTCAAGTCATGACCCTCTG**	
GFUGT2-F	**ATGGGCCAATTAGGGAGCTTAC**	To amplify the cassette containing *GFUGT88A1* for BLAST analysis
GFUGT2-R	**CTGGAGTAGGGCGACACGA**	
GLS-F	CTACGCTCCGCATAACCAACAGA	RT-qPCR analysis
GLS-R	CGCCAGCACCACTAGCAAGAA	
GMP-F	ACTCTCAAGGGTCGTTCGCTCAT	
GMP-R	GCTGCTCGGTAATGCTCTTCACA	
UXE-F	GAGTGTCGTCGCTCAACTTCCT	
UXE-R	GGTAGGTAAGCATTCGGCATTCG	
UGE-F	CGCATCCTCACATTCCTCAGCAG	
UGE-R	ATCGTCCGCCTTCGCCTCAT	
UGP-F	GCTCGCTGTGCTCAAGGTCAAT	
UGP-R	CGGTGAGGTCCAGGAAGGTCAT	
PMI-F	TCACCATTCCGCAACCACAAGT	
PMI-R	CGCAGAGACCAAGGCACCATT	
RHO1-F	GCACGTCTTGCTCTTGCTCTGA	
RHO1-R	CCGTAGTCTGTCGTACTCCTCCT	
BCK-F	GGTCCAGGAGACGAGAGCAATC	
BCK-R	CGAACGCCAGTGGTGAATCAAC	
SLT2-F	GCGAGCAGGCATTGAACCATCC	
SLT2-R	GCACGCACTTCAGCACGGAA	
