# Supplementary material for: A newly discovered glycosyltransferase gene UGT88A1 affects growth and polysaccharide synthesis of Grifola frondosa
Source: Appl Microbiol Biotechnol. 2024 Feb 29;108(1):246. doi: 10.1007/s00253-024-13062-0 (PMC10904514; doi:10.1007/s00253-024-13062-0)
Supplement: Supplementary file 1 — Supplementary file1 (PDF 598 KB) [file 253_2024_13062_MOESM1_ESM.pdf]

Applied Microbiology and Biotechnology

Supplementary Material

A newly discovered glycosyltransferase gene *UGT88A1* affects growth and polysaccharide synthesis of *Grifola frondosa*

Jian Li, Bao-xin Wang, Jie Zhang, Na Han, Shu-ting Liu, Wen-ji Geng, Shi-ru Jia, Yan-ru Li, Quan Gan, Pei-pei Han*

State Key Laboratory of Food Nutrition and Safety, Key Laboratory of Industrial Fermentation Microbiology, Ministry of Education, College of Biotechnology, Tianjin University of Science and Technology, Tianjin 300457, P.R. China

* Corresponding author: State Key Laboratory of Food Nutrition and Safety, Key Laboratory of Industrial Fermentation Microbiology, Ministry of Education, College of Biotechnology, Tianjin University of Science and Technology, Tianjin 300457, P.R. China. Email: pphan@tust.edu.cn; Tel: +86 22 60601598; Fax: +86 22 60602298

```
1       ATGGTCAGGC TGCGGGTGAT TGTGGTCACC TTCCTCACGA CGGCCGCACT TTACGATCGC
61      ATCAAGTCTG AGATATCGCG AAGCTTTGAG CCAAACGAAG AGCATCTCCT CGATCGCATT
121     AGGGTGATCG CGCTGCAGAA GAACCTTCAA GACCACTTTG GCGGAGACAC TCTTGACACT
181     GCATTTGAAG ACGCATACAG CAAGCTCATT TCGGAGGAGC CACTGACATG CGTGAAGACC
241     GGTACTTCGT ACGACTCCGT TCGCAGCCCT GATGCAGCCA TCGTGGACTT TTTCGCTATC
301     GTTCCATTCA ATGCGGCCAA AAAACTCAAC AGAAATGGCG AGGAGAGAAG GAACCTCCGT
361     GCGAAAGTGG AGGCCGAGGT CAAACGGTCC GGAAGACCTT TCAATTTGGT CGCCACGGAG
421     GTTCTCTTTG GCAGTGATGG ATCGGTCGTC CGCATTCCTG GACTGCCGCC TATGTATGAC
481     CACGAATACA ACCCGCAAGA CTTCACGTTC TCCGAAGATC TCGGTGCGAA GATACTGCTT
541     ATGACCTACG ACACGCTCGA AGCATCCGAT GGAGTCCCTC TGCTTAGCGC GGAGGCGTAC
601     GAACCAGAAT GCGTCGCCGC CGTCAGGGAA TGGTTCGCGC AGACCTCCCG CAGCGTCACC
661     GTTTGCGGAC CGCTGCTGCC CTCTGGGAAG AATGCTGCCG CTCACGAGAA GCAGCAGTCG
721     AGCGAAGGCA ACGAAATCCA GAAGTTCCTG GACTCGACCC TCGAGTCCCA CGGAACGCAT
781     TCGCTGCTTT ATATATCGTT CGGATCTATG TTCTGGCCTG CAAAGCCGGA TGTCATATGG
841     GCTTTCCTTG ACGTTGTCAT GGAGCTAAAG ATCCCCTTCA TCTTGAGCCA CGCATCGCCA
901     TTGGCCGTTG TACCTGAAGA GGTTTCCCAG AAGGTCCGCG AGTATGGACT TGGTTTACTC
961     TCACCATGGA CCCCACAGCA GACGATTCTC AACCACCCTG CGACGGGCTG GTTCGTAGCC
1021    CACGGCGGCC ACAACGGCGT GCTAGAGGCA ACAACCGCAG GCGTGCCACA AATATTCTGG
1081    CCCTTCGCCG CGGACCAGCC ACTCAATGCC GTGCACCTCA CGCACAACCT CGACGTCGCG
1141    TACGAGCTCA TCGAGGTGCG CACTGGCAAG GGGCTCTTGC CGATCCTGCG TACTGGCTAC
1201    ACACCTGTAG GCACGCCAGA GGCCGTGCGG AACGAAGCCA AGGATGTGCT CACGAAGGCA
1261    TTCGGCGAGG ACGGAGAGCG CAAGCGTGCG AAGCTGCAGG CGCTGAAAAA GGCCCTCGCG
1321    GAGTCGTGGG CGGAAGATGG CCCTTCCAGG CGTGATGTCG AAGCGCTCCT TGATTCTGTC
1381    GGTGTTTGA
```

Figure S1 CDS (coding sequence) of *UGT88A1* from *G. frondosa*

Table S1 Primers used in the present study.

Primer	Sequence (5' to 3')	Description
GFUGT-F	ATGGTCAGGCTGCGGGTGAT	Cloning *GFUGT88A1* from *G. frondosa* cDNA library
GFUGT-R	TCAAACACCGACAGAATCAAG	
OEGFUGT-F	AGTCCCTGGTAGGCAGCTTTATGGTCAGGCTGCGGGTGATT	Construction of *GFUGT88A1* overexpression plasmid with fragment
OEGFUGT-F	CGACGGCCAGTGCCAAGCTTTCAAACACCGACAGAATCAAG	
GPDA-F	AGATGCCGACCGCGGGATCCGATTTCGGCACGGCTACGGAA	Construction of *GFUGT88A1* overexpression plasmid with *gpd*A promotor
GPDA-R	**ATCACCCGCAGCCTGACCATAAAGCTGCCTACCAGGGACTG**	
**iGFUGT-F**	**ACCCACCAGTATGGTCAGGCTGCGGGTGATTG**	Construction of *GFUGT88A1* silencing plasmid
**iGFUGT-R**	**AGGCAGCTTTTCAAACACCGACAGAATCAAGGA**	
**iGPD-F**	**GGCAAAGGAATAGAGTAGATGCCGACCGCGGGATCCGTTCG**	Construction of *GFUGT88A1* silencing plasmid with *gpd* promotor
**iGPD-R**	**GCCTGACCATACTGGTGGGTACAAATGACGTC**	
**iGPDA-F**	**CGGTGTTTGAAAAGCTGCCTACCAGGGACTGA**	Construction of *GFUGT88A1* silencing plasmid with *gpd*A promotor
**iGPDA-R**	**GTTGTAAAACGACGGCCAGTGCCAAGCTGATTTCGGCACGGC**	
**HPH-F**	**CGTATATGCTCCGCATTGGTCT**	PCR verification for anti-hygromycin positive transformants
**HPH-R**	**GGCTCAAGTCATGACCCTCTG**	
GFUGT2-F	**ATGGGCCAATTAGGGAGCTTAC**	To amplify the cassette containing *GFUGT88A1* for BLAST analysis
GFUGT2-R	**CTGGAGTAGGGCGACACGA**	
GLS-F	CTACGCTCCGCATAACCAACAGA	RT-qPCR analysis
GLS-R	CGCCAGCACCACTAGCAAGAA	
GMP-F	ACTCTCAAGGGTCGTTCGCTCAT	
GMP-R	GCTGCTCGGTAATGCTCTTCACA	
UXE-F	GAGTGTCGTCGCTCAACTTCCT	
UXE-R	GGTAGGTAAGCATTCGGCATTCG	
UGE-F	CGCATCCTCACATTCCTCAGCAG	
UGE-R	ATCGTCCGCCTTCGCCTCAT	
UGP-F	GCTCGCTGTGCTCAAGGTCAAT	
UGP-R	CGGTGAGGTCCAGGAAGGTCAT	
PMI-F	TCACCATTCCGCAACCACAAGT	
PMI-R	CGCAGAGACCAAGGCACCATT	
RHO1-F	GCACGTCTTGCTCTTGCTCTGA	
RHO1-R	CCGTAGTCTGTCGTACTCCTCCT	
BCK-F	GGTCCAGGAGACGAGAGCAATC	
BCK-R	CGAACGCCAGTGGTGAATCAAC	
SLT2-F	GCGAGCAGGCATTGAACCATCC	
SLT2-R	GCACGCACTTCAGCACGGAA	
